# Model Validation for Survival Analysis by Smoothed Predictive Likelihood

**DOI:** 10.1002/sim.70193

**Published:** 2025-07-18

**Authors:** Chengyuan Lu, Hein Putter, Mar Rodríguez Girondo, Jelle J. Goeman

**Affiliations:** ^1^ Biomedical Data Sciences Leiden University Medical Center Leiden the Netherlands

**Keywords:** additive hazards, Brier score, cross‐validated partial likelihood, cross‐validation

## Abstract

Assessing the predictive performance is a crucial aspect in survival modeling, essential for model selection, tuning parameter determination, and evaluating additional predictive ability. The predictive log‐likelihood has been recommended as a suitable evaluation measure, particularly for survival models, which generally return entire survival curves rather than point predictions. However, applying predictive likelihood in semiparametric and nonparametric survival models is problematic since the survival curves are step‐functions, which result in zero predictive likelihood when events occur at previously unobserved time points. The most well‐known existing solution, Verweij's predictive partial likelihood, is limited to Cox models. In this article, we propose a novel approach based on nearest‐neighbor kernel smoothing that is usable in general semi‐ and nonparametric survival models. We show that our new method performs competitively with existing methods in the Cox setting while offering broader applicability, including testing for the presence of a frailty term and determining the optimal level of smoothness in penalized additive hazards models.

## 1 Introduction

When building survival models for prediction, it is crucial to assess predictive performance. Comparisons of predictive performance between models help in model selection [1], determining tuning parameters in penalized or regularized models [2], and assessing additional predictive ability [3]. Evaluating a model's predictive performance commonly involves fitting it to one data set and then applying it to new data, and assessing the discrepancies between the predicted and the actual outcomes. This general framework includes the often used train/test data set‐ups and cross‐validation, a repeated form of a train/test data set‐up [4]. Leave‐one‐out cross‐validation is known to be approximately unbiased for the assessment of prediction error [5] and the same holds for k‐fold cross‐validation with small k [6].

However, it is important to realize that predictions from statistical models often take the form of a predicted distribution, rather than a point prediction. This is especially true for survival models, which typically return an entire predicted survival curve (i.e., one minus a distribution function) rather than a single predicted survival time. For models returning a predictive distribution, the predictive log‐likelihood has been suggested as a suitable measure for evaluating predictions, both in statistics [7] and in machine learning, where it is known as cross‐entropy [8, 9]. The predictive log‐likelihood of an individual in the test set is the likelihood of the new observation, according to the distribution predicted for that observation. The total predictive log‐likelihood of a test set is the sum of the predictive log‐likelihoods of all the individuals in the test set.

Many popular estimation methods in survival analysis, for example for Cox models, additive hazards models [10, 11], and frailty models [12], are semiparametric or even nonparametric. Typically, these methods return a step function as an estimate of the survival curve, with steps only at the locations of the event times in the training data set used for fitting the model. The step function form leads to complications when evaluating the predictive likelihood. According to a step function survival curve, events are predicted to happen only at time points observed in the training set. Therefore, if any subject in the test set experiences an event at a different time point, the predicted likelihood for this event will be zero, regardless of the covariates or the model. As a result, the predictive likelihood of the entire test set is zero. When the true survival distribution is continuous, this unlucky event happens with probability 1. Direct application of predictive likelihood to semiparametric survival models is therefore ineffective, because all models appear equally poor.

This problem is well‐known and several solutions are used in practice. The solution of Verweij and van Houwelingen [13] is closest to the idea of predictive likelihood, replacing predictive likelihood with predictive partial likelihood. However, this solution is only suitable for Cox models, and not for general semi‐ and nonparametric contexts, which do not have an associated partial likelihood. For example, our motivating real data application concerns the smoothing via penalization of the nonparametric time‐varying regression coefficients in the context of additive hazards models, for which the predictive partial log‐likelihood is not usable.

Other approaches move away from the likelihood. The concordance index [14] only looks at relative predictions, not absolute. The Brier score (BS) [15] evaluates predictive ability at a single timepoint only, which requires an arbitrary choice of a prediction time, and reduces the prediction to a single number. The integrated BS [16] partly remedies this by averaging over many BSs. However, it has been conceptually criticized for overweighting the lower values of the survival time distribution [17], and it loses the connection to the likelihood. To the best of our knowledge, no general likelihood‐based solution exists for semiparametric models other than Cox.

In this article, we propose a novel general approach to predictive likelihood for assessing the validity of semi‐ and nonparametric survival models. We start with a detailed analysis of the solution of Verweij and van Houwelingen [13], providing an alternative interpretation of this method, showing that it “borrows” the baseline hazard from the full data set, and we argue that this approach is not easy to generalize to general semiparametric approaches. Therefore, we propose an alternative approach to solving the zero predictive‐likelihood problem, using a nearest neighbor kernel smoothing method. We compare our new method with other existing methods, including (where possible) the Verweij and van Houwelingen method and the (integrated) BS, using simulated data in the Cox model where Verweij's method can be used, and in a frailty model where it cannot. Finally, we apply the method to the problem of finding the optimal tuning parameter in a penalized nonparametric additive hazards model.

## 2 Notation and Problem Formulation

We consider the development of a semi‐ or nonparametric survival regression model M, such as a proportional hazards, frailty or additive hazards model, fitted using a training dataset ${\left(T_{i},\delta_{i},x_{i}\right)}_{i=1,\ldots ,n}$, where $T_{i}=\min({\tilde{T}}_{i},C_{i})$ is a right‐censored event time, $\delta_{i}$ is the event indicator, and $x_{i}$ is a vector of covariates of interest. We assume that the true survival time ${\tilde{T}}_{i}$ is independent of the censoring time $C_{i}$, conditionally on the vector of covariates $x_{i}$. The regression model estimates survival probabilities S(t|x) as a function of t. We assume the true survival probability curve is absolutely continuous. On the other hand, given our focus on semi‐ or nonparametric regression models, we will assume that Ŝ(t|x) is a step function, with steps only at event times in the training set.

The likelihood of such models depends only on the ordering of the event times, not on the times themselves [18]. This property is reflected in the step function estimates, which are invariant to warping the time axis. Transforming time by an increasing function, estimating, and back‐transforming will result in the same estimated curve for every transformation. This is an attractive property for survival times, which are often highly skewed.

When models provide predictive distributions, such as survival curves, the predictive log‐likelihood is the most principled loss function for evaluating predictions [7]. In the survival context, the predictive log‐likelihood is often written in terms of the hazard function, as follows:

(1) L(S(t),T,δ)=δlogh(T)−H(T)

where H(t)=−log(S(t)) is the cumulative hazard function, and h(t), the hazard, is its derivative with respect to t.

Our main focus is to assess the predictive performance of the predicted Ŝ(t|x) in a new independent test subject $\left(T_{\text{new}},\delta_{\text{new}},x_{\text{new}}\right)$. Since the hazard h(t) is 0 almost everywhere, the likelihood loss $L(Ŝ(t\;|\;x_{\text{new}}),T_{\text{new}},\delta_{\text{new}})$ given by expression (1) will be 0 in the test set observation in all cases unless $T_{\text{new}}$ happens to be an event time point in the training dataset. This makes (1) uninformative for distinguishing between competing models. To solve this problem, we need to define a new appropriate loss function $L(Ŝ(t\;|\;x_{\text{new}}),T_{\text{new}},\delta_{\text{new}})$. This loss function will allow us to quantify the discrepancy between the model's predicted survival probabilities $Ŝ(t\;|\;x_{\text{new}})$ and the actual event outcome $\left(T_{\text{new}},\delta_{\text{new}}\right)$ of the test subject.

Without loss of generality, we consider only a single test subject. If we have a test set with m independent subjects, the total loss is simply given by the sum

(2) $$\overset{m}{\underset{j=1}{\sum }}L(Ŝ(t\;|\;x_{\text{new},j}),T_{\text{new},j},\delta_{\text{new},j})$$

The set‐up also includes cross‐validation, both leave‐one‐out and K‐fold. In the case of leave‐one‐out cross‐validation, we use each individual of the dataset, one at a time, as a separate test set. We iteratively split the data into two subsets: a set comprising n−1 individuals, including all except individual i, in which we obtain a predicted curve ${Ŝ}_{i}(t\;|\;x)$, and the test set consisting of only individual i, so that $\left(T_{\text{new}},\delta_{\text{new}},x_{\text{new}})=(T_{i},\delta_{i},x_{i}\right)$. The overall leave‐one‐out cross‐validation loss is then given by:

(3) $$\overset{n}{\underset{i=1}{\sum }}L({Ŝ}_{i}(t\;|\;x_{i}),T_{i},\delta_{i})$$

The idea of K‐fold cross‐validation is similar, the data is split into K disjoint groups $D_{k}$, k=1,…,K with $D_{1}\cup \cdots \cup D_{K}=\{1,\ldots ,n\}$, of approximately equal size. We fit K models $M_{1},\ldots ,M_{K}$. For fitting model $M_{i}$, group $D_{i}$ is used as the test set and the model is trained on the remaining data. The predicted curves are ${Ŝ}_{1}(t\;|\;x),\ldots ,{Ŝ}_{K}(t\;|\;x)$, and the resulting loss function is given by

(4) $$\overset{K}{\underset{k=1}{\sum }}\underset{j\in D_{k}}{\sum }L({Ŝ}_{k}(t\;|\;x_{\text{new},j}),T_{\text{new},j},\delta_{\text{new},j})$$

Our goal is to find an alternative to the log‐likelihood loss given by expression (1) that circumvents the problem that the predictive likelihood equals 0 for an event time point from the test not seen in the training data. It should never give zero predictive log‐likelihood, but remain close to the idea of predictive log‐likelihood in spirit.

### 2.1 The Predictive Partial Likelihood in the Cox Model

In the specific context of the Cox model, a solution to our problem has been given by Verweij and van Houwelingen [13]. We will first examine their solution in more detail, to assess whether it can be generalized to other semi‐ or nonparametric survival models.

Consider a Cox model with hazard function for an individual i given by

$$h(t\;|\;x_{i})=h_{0}(t)e^{x_{i}^{⊤}\beta }$$

where $h_{0}(t)$ is an unspecified baseline hazard. Throughout, we assume there are no ties. The log‐likelihood is given by

(5) $$\ell (\beta ,h_{0}(\cdot ))=\overset{n}{\underset{i=1}{\sum }}{\left\{h_{0}(t_{i})e^{x_{i}^{⊤}\beta }\right\}}^{\delta_{i}}\exp(-H_{0}(t_{i})e^{x_{i}^{⊤}\beta })$$

The vector of regression coefficients is estimated by maximizing the *partial log‐likelihood*, given by

(6) $$\text{pl}(\beta )=\overset{n}{\underset{j=1}{\sum }}\delta_{j}\log\left(\frac{e^{x_{j}^{⊤}\beta }}{\sum_{k\in R(t_{j})}e^{x_{k}^{⊤}\beta }}\right)$$

It is well known that pl(β) can be obtained, apart from a constant term, as a profile log‐likelihood $\ell (\beta ,{ĥ}_{0}(t;\beta ))$, where ${ĥ}_{0}(t;\beta )$ is an estimate of the baseline hazard for given β, given by the hazard increment

(7) $${ĥ}_{0}(t;\beta )=\frac{1}{\sum_{j\in R(t)}e^{x_{j}^{⊤}\beta }}$$

at each event time point t.

Maximizing pl(β) leads to the maximum likelihood estimate $\hat{\beta }$, and Breslow's estimate of the baseline hazard is given by ${ĥ}_{0}(t;\hat{\beta })$. A survival curve for a subject with covariate vector x is obtained by first calculating the subject‐specific hazard $ĥ(t\;|\;x)={ĥ}_{0}(t)e^{x^{⊤}\beta }$, and then $Ŝ(t\;|\;x)=e^{-Ĥ(t\;|\;x)}$, with $Ĥ(t\;|\;x)=\sum_{t_{i}\leq t}ĥ(t_{i}\;|\;x)$, summing over all event time points $t_{i}\leq t$. When using the predictive log‐likelihood (5) as loss function, we see that $L(Ŝ(t\;|\;x_{\text{new}}),T_{\text{new}},\delta_{\text{new}})$ will be 0, if $T_{\text{new}}$ is not present in the event time points among $t_{1},\ldots ,t_{n}$ from the training data, since ${ĥ}_{0}(T_{\text{new}})=0$.

Now let us discuss cross‐validation. Suppose that we would evaluate the partial log‐likelihood for a sample with a specific subject, i, removed. When subject i is removed, the sum in (6) will exclude j=i, and subject i is also removed from all risk sets before and including $t_{i}$. Define the resulting partial log‐likelihood as ${\text{pl}}_{\left(-i\right)}(\beta )$. Then Verweij and Van Houwelingen [13] proposed to use $\text{pl}(\beta )-{\text{pl}}_{\left(-i\right)}(\beta )$ as an approximation of the individual log‐likelihood contribution of subject i, and they defined the cross‐validated partial log‐likelihood

(8) $$\text{cvpl}=\overset{n}{\underset{i=1}{\sum }}\left\{\text{pl}({\hat{\beta }}_{\left(-i\right)})-{\text{pl}}_{\left(-i\right)}({\hat{\beta }}_{\left(-i\right)})\right\}$$

where ${\hat{\beta }}_{\left(-i\right)}$ is the maximum likelihood estimator of β in the sample excluding i. This approach solves the zero predictive log‐likelihood problem, since both $\text{pl}({\hat{\beta }}_{\left(-i\right)})$ and ${\text{pl}}_{\left(-i\right)}({\hat{\beta }}_{\left(-i\right)})$ do not suffer from it.

In Appendix A, we derive a different way of viewing the cross‐validated partial likelihood. We show that each term in cvpl in Equation (8) is an approximation (using log(1+x)≈x) of a predictive log‐likelihood term $\ell ({\hat{\beta }}_{\left(-i\right)},{\tilde{h}}_{0}(t;{\hat{\beta }}_{\left(-i\right)})),$ with

$${\tilde{h}}_{0}(t;\beta )=\frac{1}{\sum_{j\in \tilde{R}(t)}e^{x_{j}^{⊤}\beta }}$$

an estimator of the baseline hazard based on the training data *including* subject i forming the test set. The risk set $\tilde{R}(t)$ includes subject i, if he/she is at risk at time t. Thus, the method of Verweij and Van Houwelingen suffers from a small amount of leakage of information from the training to the test set, in the sense that some information of the test set is included in the estimate of the baseline hazard.

Verweij's method was originally proposed for leave‐one‐out cross‐validation only. The proper extension to K‐fold cross‐validation replaces ${\hat{\beta }}_{\left(-i\right)}$ by ${\hat{\beta }}_{\left(-D_{i}\right)}$ as in formula (4). Note that this is not the same as evaluating the partial likelihood for the entire training set; doing that would violate the principle that each test set individual's contribution to the loss function may not depend on other individuals in the test set.

In summary, we see that the method of Verweij [13] relies on the existence of a partial likelihood. This implies that the approach can only be generalized to other models for which a partial likelihood can be defined. These are models in which the only nonparametric component is a nuisance parameter, such as the baseline hazard in the Cox model. Consequently, this approach is not applicable to models where the nonparametric component directly involves the estimation of the covariate effects, such as in the additive hazards model.

Moreover, the analysis in Appendix A shows that the cross‐validated partial likelihood plugs in a baseline hazard curve from an augmented data set that uses the test data. This means that the fitted model has access to some information from the test data. For proper evaluation of models, such leakage of information should be avoided at all cost. The leakage in this case could well be negligible in practice, but this has never been properly investigated.

### 2.2 A New General Approach Based on Smoothing

Since the Verweij and van Houwelingen method does not apply to general nonparametric survival models, we propose an alternative, more general, approach: establishing a log‐likelihood loss function for the test set using a smoothing method.

As previously discussed, applying expression (1) directly encounters an issue when dealing with new event times, as the hazard at these time points is zero, leading to log‐likelihood values of zero at new observations of the test set. To address this challenge, we propose a solution involving the estimation of the hazard by smoothing the estimated cumulative hazard $Ĥ(t\;|\;x_{i})$.

The estimated hazard, achieved through the smoothing of the cumulative hazard curve Ĥ(t), is defined as follows:

(9) $${ĥ}_{s}(t\;|\;x_{i})=b^{-1}\int K\left(\frac{t-s}{b}\right)dĤ(s\;|\;x_{i})$$

where K(s) is a uniform kernel function that vanishes outside [−1,1]. The predictive log‐likelihood for an arbitrary individual i in the test set then can be calculated as:

(10) $$L(Ŝ(t\;|\;x_{i}),t_{i},\delta_{i})=\delta_{i}\log({ĥ}_{s}(t_{i}\;|\;x_{i}))-Ĥ(t_{i}\;|\;x_{i})$$

A challenge of this approach is the choice of the bandwidth b. The usual rule of thumb in linear regression models, for example, is to consider a fixed bandwidth $\hat{b}=\hat{\sigma }n^{-\frac{1}{5}}$, where $\hat{\sigma }$ is the estimated error variance [19]. However, because event times in survival data typically exhibit a nonuniform distribution, with clustering at the beginning and sparsity toward the end of the support, fixed bandwidths are not advisable in our situation. Fixed bandwidths also break the valuable property of invariance to warping of the time scale that most semi‐ and nonparametric survival models have.

Therefore, we propose an adaptive bandwidth approach. We consider a bandwidth that accounts for a certain fixed number, m, of event times on each side of $t_{i}$ when estimating the hazard at point $t_{i}$, and we recommend, from practical experience, m=5. This approach essentially uses an 2m‐nearest neighbor strategy for bandwidth selection, ensuring that the estimation adapts to the distribution of event times in the training data, except that first and last few events use fewer neighbors. With this bandwidth choice, model's performance can be evaluated by incorporating the resulting expression (10) into the relevant evaluation expressions, such as (2), (3) or (4). The resulting method has the required property that it is invariant to warping of the time axis.

When applied to the Cox model, our method leads to a relatively simple expression. Since in the Cox model the hazard is defined as $ĥ(t\;|\;x_{i})={ĥ}_{0}(t)w_{i}$, it is easy to see the proportional assumption is naturally satisfied in our new method and we only need to smooth the baseline cumulative hazard ${Ĥ}_{0}(t)$:

(11) $$\begin{array}{ll} {ĥ}_{s}(t\;|\;x_{i}) & =b^{-1}\int K\left(\frac{t-s}{b}\right)dĤ(s\;|\;x_{i})=w_{i}b^{-1}\int K\left(\frac{t-s}{b}\right)d{Ĥ}_{0}(s) \end{array}$$

where $w_{i}=e^{x_{i}{\hat{\beta }}_{\left(-i\right)}}$.

In this case, the estimated cumulative baseline hazard ${Ĥ}_{0}(t)$ is a right continuous step function with finite jumps $\lambda_{i}$ at $t_{i}$ defined by Equation (7). Applying the smoothing idea to the baseline hazard, we obtain:

(12) $${ĥ}_{0s}(t)=b^{-1}\overset{n}{\underset{i=1}{\sum }}K\left(\frac{t-t_{i}}{b}\right)\lambda_{i}=b^{-1}\overset{n}{\underset{i=1}{\sum }}K\left(\frac{t-t_{i}}{b}\right)\frac{1}{\sum_{t_{l}\geq t_{i}}w_{l}}$$

and the resulting smoothed hazard in the Cox model is

(13) $${ĥ}_{s}(t\;|\;x_{i})=w_{i}{ĥ}_{0s}(t)$$

Hence, it suffices to smooth the baseline hazard function, simplifying the modeling process.

Our new smoothing method can easily fit into different types of semi‐ and nonparametric survival models, making it very flexible and widely useful. It does not have the information leak that the method of Verweij and Van Houwelingen has.

### 2.3 Penalized Additive Hazards Model

The motivating application for the development of our novel predictive‐likelihood method is tuning parameter selection in a penalized additive hazards model. The additive hazards model is a nonparametric model that assumes the hazard rate given $x_{i}^{*}={\left(1,x_{i}^{⊤}\right)}^{⊤}$ to be of the form:

(14) $$h(t\;|\;x_{i}^{*})={x_{i}^{*}}^{⊤}\beta (t)=\beta_{0}(t)+\beta_{1}(t)x_{i1}+\cdots +\beta_{p}(t)x_{ip}$$

Here, the parameters $\beta_{j}(t)$ allow effects of the covariates to change over time, making the model nonparametric per parameter. The cumulative beta $\hat{B}(t))$ is typically estimated using Aalen's ordinary least squares (OLS) estimator:

(15) $$d\hat{B}(t)=(X{\left(t\right)}^{⊤}{\left(X(t)\right)}^{-1}X{\left(t\right)}^{⊤}dN(t)$$

where N(t) is the corresponding counting process. Defining $Y_{i}(t)=I(t_{i}\geq t)$ as the at‐risk indicator of subject i, X(t) is the matrix of covariates multiplied with $Y_{i}(t)$ at the ith row. When X(t) is not of full rank, we set $d\hat{B}(t)=0$ [20]. The resulting cumulative beta $\hat{B}(t)$ is a step function, which also implies a step function for the resulting survival curve. The corresponding cumulative hazard is given by $H(t\;|\;x_{i})=\hat{B}(t)x_{i}$.

Now we introduce a penalty term to shrink the norm of the time‐dependent regression coefficients β(t), leading to the penalty term $\lambda \|\beta \|=\lambda (\sum_{i}\beta_{i}^{2})$. This aims to produce smoother cumulative beta and survival curves, enhancing both the generality and interpretability of the estimated effects. By extending the usual OLS estimator given by expression (15), we consider the following ridge penalization:

(16) $$d\hat{B}(t)={\left(X{\left(t\right)}^{⊤}X(t)+\lambda I\right)}^{-1}{\left(X(t)\right)}^{⊤}dN(t)$$

where we keep $d\hat{B}(t)=0$ if X(t) is not of full rank, as above.

The penalty λ‖β‖ serves the purpose of moderating the magnitude of β. By doing so, the survival curve will be smoother and mitigate excessive fluctuations. Different Lagrange multiplier values result in different β estimations and consequently distinct prediction error estimations. We can identify the λ value that yields the minimal prediction error by minimizing the cross‐validation error using our new approach based on smoothed predictive likelihood.

## 3 Simulation Studies

We conducted two simulation studies to investigate the practical performance of our new approach to model selection. In the first simulation, we investigated variable selection within the framework of the Cox model. In this setting, we were able to compare with the approach of Verweij and Van Houwelingen, as well as with BS and the integrated Brier score (IBS). The second setting focused on model specification assessment, specifically choosing between a frailty model and a Cox model. This is a context in which the approach of Verweij and Van Houwelingen cannot be used.

### 3.1 Simulation 1: Variable Selection in the Cox Model

As a first proof‐of‐principle experiment, we investigate whether the predictive likelihood from our new smoothing approach can accurately select the correct Cox model from a large pool of models that include noise variables, particularly in the context of a limited sample size. We compare its performance against three alternative approaches.

We generated data using a Cox model across three scenarios. In each scenario, the true model included one, two, or three variables, accompanied by three, two, or one noise variables, respectively. Thus, there were always four possible variables and $2^{4}=16$ candidate models for selection, formed by all possible combinations of inclusion and exclusion of the four variables. Variables $x_{1}$ to $x_{4}$ were generated independently following a standard normal distribution, and the true Cox model was defined by $h(t)=h_{0}(t)e^{\sum_{i}\beta_{i}x_{i}}$, with constant baseline hazard $h_{0}=1$. The size of β was varied to reflect different levels of signal‐to‐noise ratio. The right censoring time was generated according to a uniform distribution on [0,C], where C was chosen such that 40% of the data were censored. We considered a sample size of n=500. Our goal was to identify the optimal model from the 16 candidate models.

We used 10‐fold cross‐validation to calculate the prediction error according to four loss functions: the new smoothing likelihood, Verweij's profile likelihood, the BS, and the IBS. For the BS, the median of the survival times serves as the event time to take value. Both the Brier score and the integrated Brier score were calculated with inverse probability weighting using a Kaplan–Meier estimate of the censoring distribution. For the smoothing likelihood, we used a 10‐nearest neighbors approach, considering, for every event point in the validation set, the closest five adjacent event times at each side. In our main analysis, for each loss function, the best‐performing model was defined as the one with the lowest prediction error. Additionally, we considered a second optimal model definition based on the widely used “one standard error rule”. This rule defines the optimal model as the most parsimonious model whose error is no more than one standard error worse than the error of the best model, that is, the model with the largest cross‐validated likelihood [1][page 216]. The one‐standard error rule was designed to counter the bias of cross‐validation toward too large models and tends therefore to lead to smaller models. As the performance measure, we reported the frequency with which the true model was selected as the optimal model across M=1000 Monte Carlo replications.

**FIGURE 1:**
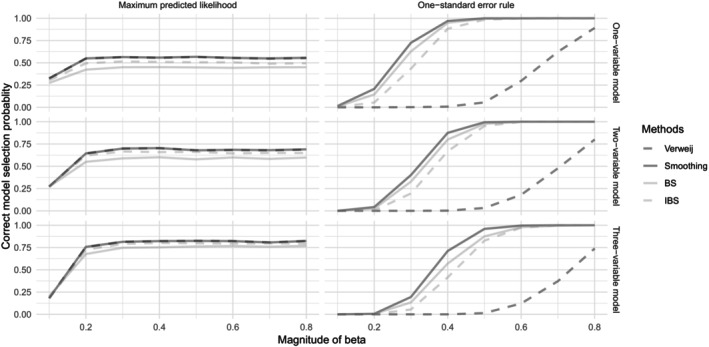
Simulation 1. Correct model selection frequencies over M=1000 Monte Carlo trials for different levels of covariate effects (β). Left panel: model selection based on the maximum predicted likelihood. Right panel: model selection based on the one‐standard error rule criterion. The top row shows results for the scenario where the true model contains one covariate and three noise variables, the middle row assumes a true model with two covariates and two noise variables, and the bottom row shows results for the scenario with a true model containing three covariates and one noise variable.

Figure 1 summarizes the results of this simulation. The top panels show the results for the first scenario with a one‐variable true model and three noise variables; the middle panels show the results for the second scenario involving a two‐variable true model, and the bottom panels refer to the third scenario with a three‐variable true model and one noise variable. Left panels show the results using the minimum prediction error as the optimal model definition, and right panels display the results according to the “one standard error rule”. In each graphic, the correct model selection frequency across the M=1000 Monte Carlo trials is displayed for each of the four competitor methods across a grid of increasing values of the non‐zero regression coefficient β and for a censoring level of 40%.

Figure 1 demonstrates that all four considered methods perform better with increasing magnitude of β and with a higher signal–noise ratio. Comparing left to right panels, no optimality rule—whether minimizing prediction error or using the one standard error rule—dominates the other. However, the one standard error rule is generally preferred in scenarios with a low signal–noise ratio, provided the regression coefficient is not too low.

The performance of the likelihood‐based methods, Verweij's method, and our new smoothing approach are virtually identical when using the criterion of minimizing prediction error and emerge as the optimal choices. The IBS exhibits a slightly worse performance when the signal‐to‐noise ratio is moderate or high (left panels, middle and bottom graphics) but clearly worse than the predictive‐likelihood approaches in the scenario of low signal‐to‐noise ratio. The performance of the BS is worse than its competitors in all studied scenarios. Notably, when considering the one standard error rule, the smoothing likelihood method is consistently the best‐performing method and slightly outperforms both the BS and the IBS, while Verweij's method performs significantly worse. This poor performance is due to Verweij's method being sensitive to the level of censoring; the variation in censorship across folds increases the standard error, masking the advantage of the true model's likelihood.

### 3.2 Simulation 2: Selecting Between Frailty and Cox Model

In this second simulation setting, we show the effectiveness of our new method based on smoothed likelihood for selecting the most suitable semiparametric survival regression model. Specifically, we focus on the choice between frailty and Cox models. This is a setting in which the method of Verweij and van Houwelingen cannot be used.

A frailty model is characterized by its hazard function represented as h(t|Z)=Zh(t), where Z denotes an unobserved individual‐specific random effect term known as frailty, and $h(t)=h_{0}(t)\exp(\beta x)$ is a multiplicative hazard function. To ensure identifiability, we assume that the expected value of frailty, denoted as E(Z), equals 1. Furthermore, we assume that frailty Z follows a gamma distribution with a variance of θ. Within this framework, we consider several scenarios where our true model is a frailty model with increasing frailty variance, including the zero variance case that represents the Cox model. The task is correctly to select between Cox and frailty model using the 10‐fold cross‐validation likelihood, computed using the new smoothing approach, as loss function. The cross‐validation log‐likelihood for the Cox model is obtained using the combination of expressions (10) and (13). The log‐likelihood of individual i for the frailty model is given by:

(17) $$L(Ŝ(t\;|\;x_{i}),t_{i},\delta_{i})=\delta_{i}\log({ĥ}_{m}(t_{i}\;|\;x_{i}))-H_{m}(t_{i}\;|\;x_{i})$$

where $H_{m}(t)$ is defined as $-\log(S_{m}(t))$, and $S_{m}(t)=E_{Z}e^{-ZH(t)}$ is the marginal survival probability, which can be obtained by integrating the conditional survival function with respect to the distribution of the frailty Z. As usual, $h_{m}(t)$ is the derivative of the function $H_{m}(t)$.

Equation (9) can be applied to estimate ${ĥ}_{m}(t_{i})$ and obtain a smoothed, predictive version of expression (17). In practice, we used the R package frailtyEM to obtain all necessary information to obtain the predictive frailty likelihood using expression (17).

When θ=0, the frailty model is equivalent to the Cox model. In this case, the marginal survival probability is the same as the conditional survival probability and the marginal likelihood Equation (17) is equivalent to the smoothing likelihood Equation (10) derived for the Cox model.

For the smoothing method, we used the nearest neighborhood method with m=5, which means we considered five time points on each side to estimate the hazard for a given time point. We used a sample size of n=500, and a censoring rate of about 40%. These settings are the same as in the first simulation. The true model was $h(t\;|\;Z)=Ze^{\beta x}$ with only one covariate and a true value of β=0.8. We used 10‐fold cross‐validation of the proposed smoothed likelihood to evaluate the performance of the models. Additionally, we compared our new approach with two other loss functions: the BS and the IBS, also calculated using 10‐fold cross‐validation. Note that the Verweij and van Houwelingen approach is not applicable in this setting since it cannot be applied to the frailty model. The results are presented in Table 1.

**TABLE 1:** Simulation 2. Model selection frequency of the frailty model over M=1000 Monte Carlo replications for increasing values of the frailty variance θ.

θ	Smoothed likelihood	Brier score	Integrated Brier score
0	44%	93%	41%
0.01	30%	83%	43%
0.1	46%	85%	38%
0.5	56%	90%	72%
1	89%	50%	100%
2	98%	3%	40%
3	97%	12%	2%
5	98%	32%	0%

Table 1 shows the selection frequency across the M=1000 Monte Carlo replications of each model according to each of the considered loss functions across varying levels of simulated frailty variance. We expect an increasing selection frequency of selection of the frailty model as the level of the frailty variance increases. When the frailty variance is zero, there is no difference between Cox model and frailty model. In this case, we expect to see a balance of selection between Cox model and frailty model. The results show this pattern for our new method based on smoothing, with an increasing selection frequency of the frailty model as the level of frailty variance increases. When the simulated frailty variance is 0 or close to 0, the new method accordingly shows a selection frequency around 50% for both models. The performance of the IBS is comparable to the smoothed likelihood when frailty variance levels are low or moderate, but it exhibits erratic behavior, with both high and low selection frequencies for the Cox model when the underlying frailty variances are large. The BS shows a systematic preference for the frailty model at low frailty variance, with the same erratic behavior as the IBS for larger variance.

## 4 Real Data Illustration: Smoothing in the Additive Hazards Model

In this application, we demonstrate the use of the new smoothed likelihood to perform penalization in the additive hazards model [10, 11]. This serves as a proof‐of‐principle, and to illustrate how our approach could be used in realistic semi‐ and nonparametric survival regression settings. Our specific goal is to use the cross‐validated likelihood obtained with our new approach based on smoothing to determine the optimal level of penalization in expression (16), leading to an optimal trade‐off between flexibility and smoothness and avoiding the excessively wiggly time‐dependent regression coefficients directly obtained from the nonparametric fit given by expression (15).

We used data from a clinical trial involving 195 patients diagnosed with oropharynx carcinoma, conducted by the Radiation Therapy Oncology Group in the United States. The dataset was introduced in the book by Kalbfleish & Prentice [21] and it was previously used in the context of the additive hazards model [20]. We consider survival time, measured in days from diagnosis, as time to event of interest and we considered seven covariates of interest: Sex, Treatment, Grade, Age, Condition, T‐stage, and N‐stage. Our aim is to apply the additive hazards model (14) with these seven covariates, while regularizing the norm of the time‐dependent coefficients β(t), leading to the ridge estimator given in Equation (16).

**FIGURE 2:**
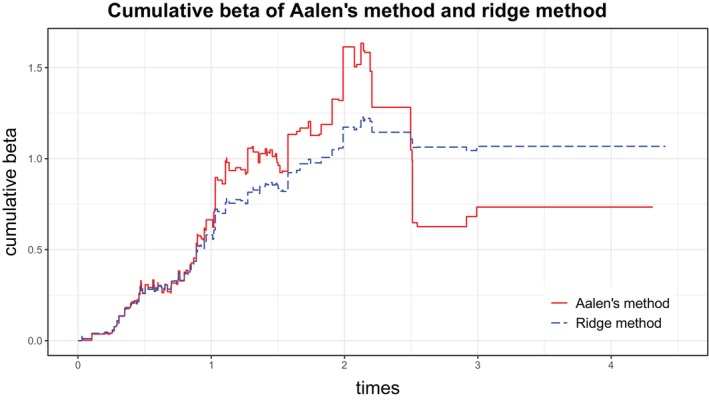
Real data application. Cumulative beta from the additive hazards model for an average individual, estimated with the Aalen's method and the new ridge approach. The optimal ridge penalty ($\lambda =10^{0.9}$) was determined using the newly developed smoothed likelihood approach.

A grid of 30 values between 0 and 3 for the logλ was considered to determine the optimal penalty parameter λ. The cross‐validation curve with respect to logλ was concave, and the maximum value of predictive likelihood was achieved at the value logλ=0.9. Figure 2 shows the estimated cumulative beta with the usual Aalen's method and the ridge approach with the optimal penalty parameter $\lambda =10^{0.9}$ obtained with the new approach based on smoothed likelihood. These curves can also be regarded as the cumulative hazard for an individual with all considered covariates taking the average value. The effect of the penalty is apparent, resulting in a smoother curve with the ridge approach. The difference between the unpenalized and penalized curves is especially notable with increasing scarcity of event times at later follow‐up time points. Note that the well‐known problem of decreasing cumulative hazard estimates for individuals based on ordinary least squares is mitigated using ridge regression, because of the implied smoothing, but it is still present. In [22] we proposed a constrained maximum likelihood method to remove this problem. It would be of interest to combine our maximum likelihood method with penalization. This is planned as future work.

## 5 Discussion

In this article, we have introduced a novel approach for calculating predictive likelihood within the context of semiparametric and nonparametric survival regression models. The key innovation of this approach lies in the use of smoothing techniques to solve the problems posed by the null hazards inherent in the step survival functions of these models. An important asset of our method is that it retains the property that it is invariant to transformation of the time scale.

Furthermore, we have revisited the classical Verweij and van Houwelingen approach to cross‐validation in the Cox proportional hazards model, clarified its connection with the leave‐one‐out cross‐validation framework when considering the partial likelihood as the loss function. These insights have provided a broader perspective on this well‐established method. In particular, we have shown that the method “borrows” the baseline hazard from the full data, which potentially leaks some information between training and test sets.

Our first, preliminary empirical investigations have demonstrated the usefulness of our proposed approach for various purposes. Within the context of Cox models, we have shown it to be competitive with the Verweij and van Houwelingen method for variable selection. Moreover, our method consistently outperformed alternative loss functions not based on likelihood, supporting its utility for predictive performance evaluation. Finally, we have shown the potential of this new approach for performing penalization in the context of semi‐ and nonparametric survival regression models. In particular, we have used the new smoothing method for deriving smoother time‐dependent covariate effects in the context of the additive hazards model. To our knowledge, no alternative likelihood‐based method exists for this purpose. In the context of model misspecification, it should be noted that the “least false” model chosen by our method will depend on the censoring distribution. However, an advantage of our approach is that this censoring distribution does not have to be estimated, thereby avoiding the risk of additional misspecification.

For the smoothing in our method, we have advocated a fixed number of m=5 neighboring time points on both sides for small to medium‐scale survival analysis problems. This was a heuristic choice. A more principled approach to bandwidth selection is likely to require that the number of neighbors should grow with the sample size at a slow rate in n to achieve consistency. In some models, the bandwidth could be allowed to depend on covariates. We did not go into the asymptotic theory of bandwidth selection in this article. Further research, theoretical and empirical, is needed to establish the properties of our proposed method.

## Disclosure

The authors have nothing to report.

## Conflicts of Interest

The authors declare no conflicts of interest.

## Supporting information


**Data S1.** Supporting Information.

## Data Availability

The data that support the findings of this study are available in The Comprehensive R Archive Network at https://cran.r‐project.org. These data were derived from the following resources available in the public domain: – CRAN R package invGauss, https://cran.r‐project.org/web/packages/invGauss/index.html.

## Appendix A The Predictive Partial Likelihood in the Cox Model

Let us start with a Cox model whose hazard function for an individual i is given by

$$h(t\;|\;x_{i})=h_{0}(t)w_{i}$$

where $h_{0}(t)$ is the baseline hazard and $w_{i}=w_{i}(\beta )$ is the relative risk defined by

$$w_{i}=e^{x_{i}^{⊤}\beta }$$

Assume there are no ties, and sort the observed time points in ascending order. Define the (also sorted) event time points ($t_{i}$'s for which $\delta_{i}=1$) as $t_{k}^{*}$, k=1,…,D, with D the total number of events in the data. Define $H_{0}(t)=\int_{0}^{t}h_{0}(s)ds$ to be the cumulative baseline hazard. The maximum likelihood estimator of ${Ĥ}_{0}(t)$ is a step function with jumps $\lambda_{k}$ at event time point $t_{k}^{*}$. The estimated $Ŝ(t)=e^{Ĥ(t)}$. The log‐likelihood of the data associated with the Cox model is given by

(A1)
$$\begin{array}{ll} \overset{n}{\underset{i=1}{\sum }}L(Ŝ(t),t_{i},\delta_{i}) & =\overset{n}{\underset{i=1}{\sum }}\left\{\delta_{i}(\log h(t_{i}))-\log Ŝ(t_{i})\right\}\\ & =\overset{n}{\underset{i=1}{\sum }}\left\{\delta_{i}(\log w_{i}+\log\lambda_{k(i)})-w_{i}\underset{t_{k}^{*}\leq t_{i}}{\sum }\lambda_{k}\right\} \end{array}$$

Here k(i) is the index among the event time points for which $t_{k(i)}^{*}=t_{i}$. We may treat this function of variable β and $\lambda_{i}$ and denote the log‐likelihood by $\ell (\beta ,\{\lambda_{k}\})$. For many purposes it is easier to rearrange (A1) as a sum over the event time points, leading to

(A2)
$$\ell (\beta ,\{\lambda_{k}\})=\overset{D}{\underset{k=1}{\sum }}\left\{\log w_{i(k)}+\log\lambda_{k}-\lambda_{k}\underset{l\in R(t_{k}^{*})}{\sum }w_{l}\right\}$$

with i(k) is the index among the individuals for which $t_{i(k)}=t_{k}^{*}$. Cox's partial likelihood can be derived as a profile likelihood, profiling out the baseline hazard: for fixed β, first maximize (A2) with respect to each of the $\lambda_{k}$'s. This yields the Breslow estimate of the baseline hazard

(A3)
$${\hat{\lambda }}_{k}(\beta )=\frac{1}{\sum_{l\in R(t_{k}^{*})}w_{l}}$$

Putting ${\hat{\lambda }}_{k}(\beta )$ back for $\lambda_{k}$ into (A2) gives the profile log‐likelihood

(A4)
$$\ell (\beta ,\{{\hat{\lambda }}_{k}(\beta )\})=\overset{D}{\underset{k=1}{\sum }}\left\{\log\left(\frac{w_{i(k)}}{\sum_{l\in R(t_{k}^{*})}w_{l}}\right)-1\right\}$$

Ignoring the (−1) terms in the profile log‐likelihood gives Cox's partial log‐likelihood given by

(A5)
$$\text{pl}(\beta )=\overset{D}{\underset{k=1}{\sum }}\log\left(\frac{w_{i(k)}}{\sum_{l\in R(t_{k}^{*})}w_{l}}\right)=\overset{n}{\underset{j=1}{\sum }}\delta_{j}\log\left(\frac{w_{j}}{\sum_{l\in R(t_{j})}w_{l}}\right)$$

Now we turn to cross‐validation. Suppose that we would evaluate the partial log‐likelihood for a sample with a specific subject, i, removed. When subject i is removed, the last sum in (A5) will exclude j=i, and subject i is also removed from all risk sets before and including $t_{i}$. Define the resulting partial log‐likelihood as ${\text{pl}}_{\left(-i\right)}(\beta )$. Then [13] proposed to use $\text{pl}(\beta )-{\text{pl}}_{\left(-i\right)}(\beta )$ as an approximation of the individual log‐likelihood contribution of subject i, and they defined the cross‐validated partial log‐likelihood

(A6)
$$\text{cvpl}=\overset{n}{\underset{i=1}{\sum }}\left\{\text{pl}({\hat{\beta }}_{\left(-i\right)})-{\text{pl}}_{\left(-i\right)}({\hat{\beta }}_{\left(-i\right)})\right\}$$

where ${\hat{\beta }}_{\left(-i\right)}$ is the maximum likelihood estimator of β in the sample excluding i. We will show that $\text{pl}(\beta )-{\text{pl}}_{\left(-i\right)}(\beta )$ can indeed be seen as an approximation of the individual log‐likelihood contribution of subject i, that is, $\ell_{i}(\beta ,\{\lambda_{k}\})$ of Equation (A1), but using for $\left\{\lambda_{k}\right\}$ the Breslow estimates from Equation (A3), based on all observations, including subject i itself. Thus, in the ith summand, $\text{pl}({\hat{\beta }}_{\left(-i\right)})-{\text{pl}}_{\left(-i\right)}({\hat{\beta }}_{\left(-i\right)})$ in cvpl uses ${\hat{\beta }}_{\left(-i\right)}$ as estimate of β, but Breslow's estimate of the baseline hazard based on the *full* data. This avoids the problem of zero likelihood for any new data point with an event time not seen in the data so far, but it also implies some data leakage, because some data of subject i is used in the validation of subject i.

To see why this approximation holds, consider $\text{pl}(\beta )-{\text{pl}}_{\left(-i\right)}(\beta )$ for subject i. If $t_{i}$ is not an event time, then the sum on the right‐hand side of (A5) is over the same subjects, whether or not we include subject i, so the only difference is in the risk sets. This difference is only there for $t_{j}\leq t_{i}$. Hence we obtain

(A7)
$$\begin{array}{ll} & \text{pl}(\beta )-{\text{pl}}_{\left(-i\right)}(\beta )\\ & \;=\underset{j:t_{j}\leq t_{i}}{\sum }\delta_{j}\left\{\log\left(\frac{w_{j}}{\sum_{l\in R(t_{j})}w_{l}}\right)-\log\left(\frac{w_{j}}{\sum_{l\in R(t_{j}),l\neq i}w_{l}}\right)\right\}\\ & \;=\underset{k:t_{k}^{*}\leq t_{i}}{\sum }\log\left(\frac{\sum_{l\in R(t_{k}^{*}),l\neq i}w_{l}}{\sum_{l\in R(t_{k}^{*})}w_{l}}\right)=\underset{k:t_{k}^{*}\leq t_{i}}{\sum }\log\left(1-\frac{w_{i}}{\sum_{l\in R(t_{k}^{*})}w_{l}}\right) \end{array}$$

If $t_{i}$ is an event time, say $t_{k}^{*}$, then we have the same difference between pl(β) and ${\text{pl}}_{\left(-i\right)}(\beta )$ as in Equation (A7), but in addition there is a contribution $\log w_{i}+\log\lambda_{k}=\log\left(\frac{w_{i}}{\sum_{l\in R(t_{k}^{*})}w_{l}}\right)$. Combining both options, we see that

(A8)
$$\begin{array}{ll} \text{pl}(\beta ) & -{\text{pl}}_{\left(-i\right)}(\beta )=\delta_{i}\log\left(\frac{w_{i}}{\sum_{l\in R(t_{i})}w_{l}}\right)+\underset{k:t_{k}^{*}\leq t_{i}}{\sum }\log\left(1-\frac{w_{i}}{\sum_{l\in R(t_{k}^{*})}w_{l}}\right) \end{array}$$

Looking on the other hand at the log‐likelihood contribution of subject i, using Equation (A1), we see that it equals

$$\delta_{i}(\log w_{i}+\log\lambda_{k(i)})-w_{i}\underset{t_{k}^{*}\leq t_{i}}{\sum }\lambda_{k}$$

Replacing $\lambda_{k}$ by ${\hat{\lambda }}_{k}(\beta )=\frac{1}{\sum_{l\in R(t_{k}^{*})}w_{l}}$, the Breslow estimator of the baseline hazard based on all observations, gives the contribution

(A9)
$$\delta_{i}\log\left(\frac{w_{i}}{\sum_{l\in R(t_{i})}w_{l}}\right)-w_{i}\underset{t_{k}^{*}\leq t_{i}}{\sum }\frac{1}{\sum_{l\in R(t_{k}^{*})}w_{l}}$$

If we use the approximation log(1+x)≈x for small x, we see that Equation (A8) is indeed an approximation of Equation (A9).
